# Spark Plasma Sintering of Lithium Aluminum Germanium Phosphate Solid Electrolyte and its Electrochemical Properties

**DOI:** 10.3390/nano9081086

**Published:** 2019-07-29

**Authors:** Hongzheng Zhu, Anil Prasad, Somi Doja, Lukas Bichler, Jian Liu

**Affiliations:** School of Engineering, Faculty of Applied Science, The University of British Columbia, Kelowna, BC V1V 1V7 Canada

**Keywords:** spark plasma sintering, NASICON-type, ionic conductivity, solid electrolyte, solid–solid interface, grain-boundary resistance

## Abstract

Sodium superionic conductor (NASICON)-type lithium aluminum germanium phosphate (LAGP) has attracted increasing attention as a solid electrolyte for all-solid-state lithium-ion batteries (ASSLIBs), due to the good ionic conductivity and highly stable interface with Li metal. However, it still remains challenging to achieve high density and good ionic conductivity in LAGP pellets by using conventional sintering methods, because they required high temperatures (>800 °C) and long sintering time (>6 h), which could cause the loss of lithium, the formation of impurity phases, and thus the reduction of ionic conductivity. Herein, we report the utilization of a spark plasma sintering (SPS) method to synthesize LAGP pellets with a density of 3.477 g cm^−3^, a relative high density up to 97.6%, and a good ionic conductivity of 3.29 × 10^−4^ S cm^−1^. In contrast to the dry-pressing process followed with high-temperature annealing, the optimized SPS process only required a low operating temperature of 650 °C and short sintering time of 10 min. Despite the least energy and short time consumption, the SPS approach could still achieve LAGP pellets with high density, little voids and cracks, intimate grain–grain boundary, and high ionic conductivity. These advantages suggest the great potential of SPS as a fabrication technique for preparing solid electrolytes and composite electrodes used in ASSLIBs.

## 1. Introduction

The utilization of rechargeable lithium-ion batteries (LIBs) has been increasingly expanded from consumer devices to plug-in or hybrid electric vehicles and smart grid energy storage systems, in order to alleviate the dependence on fossil fuels, decrease greenhouse gas emissions, and realize clean transportation [1,2]. However, traditional LIBs utilize liquid organic electrolytes that could cause catastrophic disasters (such as fire and explosion) upon exposure to the air due to their flammable and volatile nature [3,4,5]. Moreover, the energy densities of current LIBs are gradually approaching their theoretical limits and cannot meet the increasing demands from end users for higher-performance LIBs. From this aspect, all-solid-state lithium-ion batteries (ASSLIBs) have recently been revisited as promising next-generation battery systems because of their significantly improved energy density and safety [3,6,7,8]. The major difference between ASSLIBs and conventional LIBs is the use of solid-state electrolytes (SSEs), rather than liquid organic electrolytes, in the design and fabrication of battery systems. The adoption of SSEs not only can significantly reduce the safety risks associated with the flammable, volatile, and toxic liquid organic electrolytes, but also could potentially address the Li dendrite growth problem and make it possible for the utilization of Li metal with a high theoretical capacity of 3860 mA h g^−1^ as the anode, therefore substantially elevating energy densities and the safety of ASSLIBs.

Among different kinds of SSEs, including garnet [9,10], perovskite [11,12], sulfides [13], hydrides [14], borate or phosphate [15,16,17], halides [18], lithium phosphorousoxynitride (LiPON) [19], lithium superionic conductor (LISICON) [20,21,22,23], and other Li-based ceramic [24], sodium superionic conductor (NASICON)-type is one of the most popular solid electrolytes, due to its high ionic conductivity and good chemical and thermal stability with lithium anode [25,26,27,28]. In particular, lithium aluminum germanium phosphate (LAGP) has a NASICON-type structure and possesses several advantages for material preparation and practical applications in ASSLIBs. First, the high stability of LAGP SSEs against O_2_ and H_2_O means that the synthesis of materials and the assembly of batteries could be performed in an ambient atmosphere, therefore simplifying the manufacturing processes and requirements [3,4,5]. Secondly, the predominant ionic conductivities of LAGP SSEs are in the order of 10^−3^–10^−5^ S cm^−1^ at room temperature (RT), which are relatively high compared with other ceramic electrolytes [29,30]. Thirdly, LAGP SSEs exhibit a large electrochemical stability window (1.8−7 V vs. Li^+^/Li), good chemical compatibility with cathode materials at different charge states, and excellent interfacial stability towards Li metal anode [6,31,32]. Nevertheless, it was also reported that short-circuit occurred in SSEs because the deposited lithium grew along voids among grains, and an effective approach to suppress lithium dendrites was to enhance the density and the mechanical strength of SSEs and create stable grain–grain interfaces in the solid electrolytes [33]. Therefore, it is imperative to develop methods to fabricate SSE pellets with high density and free of voids and through-holes. However, conventional sintering methods usually require high temperatures (>800 °C) and long sintering time (>6 h), which could easily result in grain coarsening, formation of impurity phases, and thus reduced ionic conductivity [34].

In recent years, spark plasma sintering (SPS) is attracting increasing attention from researchers to fabricate solid electrolytes and electrodes with the densities close to their theoretical values in a short sintering time [35,36]. During SPS processes, uniaxial force and pulsed direct electrical current are simultaneously applied to the powders and thus can rapidly consolidate the powders into dense pellets. The use of microscopic electrical current allows the sintering to complete in a few minutes, because of the very high heating rates and the localized high temperatures between particles. In the meantime, the water-cooling system in SPS allows a very high cooling rate. Compared to the conventional heat treatment, the effective heating and cooling systems in SPS enhanced the densification of SSE powders through grain diffusion mechanisms and avoided grain coarsening to maintain the intrinsic merits of nano-powders [37,38]. Indeed, SPS technique, with the advantages of a flash and short processing time, improves the sintering ability of various powder materials and creates intimate solid–solid interfaces in solid electrolytes and electrodes for ASSLIBs [30,39].

In this work, we have successfully produced high-densified LAGP pellets, with a high ionic conductivity of 3.29 × 10^−4^ S cm^−1^ by using SPS. Owing to the advantage of minimum energy and time consumptions of SPS, the highly dense LAGP without microcracking resulted in the reduced resistance at grain boundaries, due to the removal of the pores/voids/cracks at a proper sintering temperature, thus improving the overall ionic conductivity of LAGP. At the same time, the low operating SPS temperature avoided the formation of ionic nonconductive impurities, which usually appeared in the grain boundaries for traditionally high-temperature sintered samples and resulted in blocking of Li-ion transport pathways.

## 2. Materials and Methods

LAGP powders with a stoichiometric formula of Li_1.5_Al_0.5_Ge_1.5_(PO_4_)_3_ were placed in a graphite tooling (1 mm die) of the SPS chamber (Thermal Technologies 10-3 SPS system) and heated up to the target sintering temperature in argon atmosphere. The SPS sintering pressure was 60 MPa, the sintering temperatures were changed from 600 °C to 750 °C, and the sintering time was varied between 1 min and 10 min for all the experiments. The SPS heating rate was set to 100 °C min^−1^. Upon the completion of SPS sintering, the samples were cooled down to room temperature (RT) naturally. In addition to the SPS, LAGP pellets were also made by a conventional dry-pressing method, where 0.5 g of LAGP powder was placed into a stainless steel die with 10 mm diameter and pressed at 200 MPa for 2 min. The obtained pellets were then placed into a tube furnace and annealed at 800 °C for 6 h under nitrogen atmosphere.

The phase structure of the starting LAGP powders and the LAGP pellets were characterized by X-ray diffraction (XRD, D8-Advance X-ray diffractometer, Bruker, Karlsruhe, Germany) with Cu K_α1_ and K_α2_ radiation source. The LAGP microstructure was observed by field-emission scanning electron microscopy (MIRA3 FEG-SEM, Tescan, Brno, Czech Republic). The density of the LAGP pellets was measured by the Archimedes’ method. For ionic conductivity measurement, the surfaces of the LAGP pellets were first polished with sandpapers to remove carbon residual from the graphite die during the SPS process, and then coated with 100 nm Au layers on both sides as blocking electrodes. An electrochemical impedance spectroscopy (EIS) test of the Au-coated LAGP pellets was conducted on a Potentiostat/Galvanostat Station (Biologic VSP) in a frequency range of 0.1 Hz–1 MHz at different temperatures of –10 °C to 80 °C.

## 3. Results and Discussion

**Figure 1:**
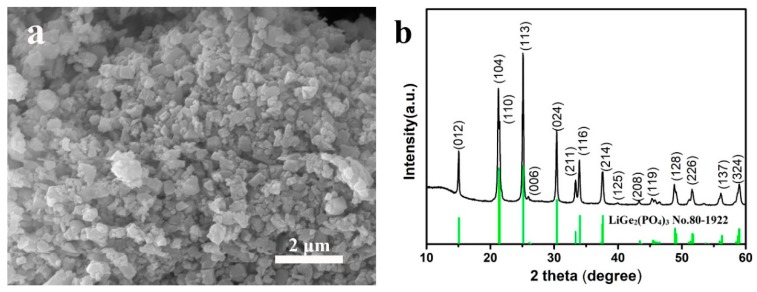
(**a**) SEM image and (**b**) XRD pattern of lithium aluminum germanium phosphate (LAGP) powders.

As the starting material, LAGP powders were characterized by SEM for the morphology and by XRD for the phase structure, and the results are presented in Figure 1. As shown in Figure 1a, the diameter of LAGP particles was about 400–800 nm. The XRD pattern of LAGP powders (Figure 1b) shows strong diffraction peaks, which could be well indexed to LiGe_2_(PO_4_)_3_ with a NASICON-type structure (JCPDS PDF No. 80-1922). No other peaks of impurities are observed in the XRD pattern, indicating the high purity of the starting LAGP powders.

**Figure 2:**
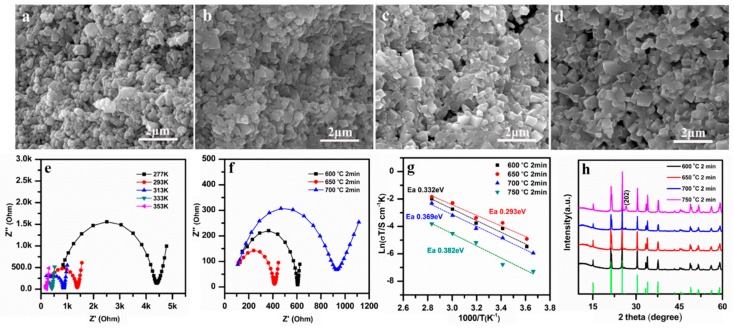
SEM images at cross sections of the LAGP pellets sintered by spark plasma sintering (SPS) for 2 min at (**a**) 600 °C, (**b**) 650 °C, (**c**) 700 °C, and (**d**) 750 °C; (**e**) Nyquist plots of the LAGP pellet, sintered at 650 °C for 2 min; (**f**) Nyquist plots at room temperature (RT), (**g**) Arrhenius curves, and (**h**) XRD patterns of LAGP pellets, sintered by SPS for 2 min at 600 °C, 650 °C, 700 °C, and 750 °C.

The effects of SPS sintering temperatures (600–750 °C) on the morphology, ionic conductivity, and phase structure of LAGP pellets was studied first. Figure 2a–d presents the SEM images of the cross-sectional view of LAGP pellets sintered by SPS at different temperatures of 600 °C, 650 °C, 700 °C, and 750 °C for 2 min. It can be observed that the grain size in all the LAGP pellets was below 800 nm, similar to that of the starting LAGP powders (Figure 1a). The LAGP grain size remained almost unchanged at sintering temperatures of 600–750 °C, due to short SPS sintering time (2 min). As seen in Figure 2a,b, LAGP pellets fabricated at 600 °C and 650 °C were dense with minimal visible porosity. In contrast, the sample sintered at 700 °C in Figure 2c exhibited enlarging pores at grain boundaries, and the one sintered at 750 °C in Figure 2d possessed large cracks in the pellets’ cross section, as seen in Figure S1. The high temperature at 750 °C possibly resulted in rapid grain growth, leading to the relatively high thermal expansion anisotropy of LAGP. Thus, the growing pores and voids likely coalesced and gradually emerged on the sample’s surfaces.

The Nyquist plots of the LAGP pellets were measured by EIS and fitted by using an equivalent circuit of *R*_1_(*R*_2_*∥*CPE)W to obtain their ionic conductivities. As illustrated in Figure 2e,f, a typical Nyquist plot consists of one semicircle in the high-frequency region and a large spike in the low-frequency region. In general, the intercept of the high-frequency semicircle with the real axis stands for the total resistance of *R*_2_ = (*R*_bulk_ + *R*_interface_), where *R*_bulk_ and *R*_interface_ represent the bulk resistance and grain-interface resistance, respectively. The spike in the low-frequency domain is known as Warburg diffusion impedance, which is due to the polarization resulting from Li-ion conduction at the electrolyte/Au electrode interface [40]. Figure 2e shows one EIS example of LAGP sintered at 650 °C, measured at different temperatures between –10 °C and 80 °C. The complex Nyquist plots of the LAGP pellets prepared between 600 °C and 750 °C are shown in Figure 2f. As supported by the SEM images (Figure 2c,d, and Figure S1), the appearance of voids and cracks in the LAGP pellets sintered at high temperatures (700 °C and 750 °C) likely caused the increase in the resistance and the reduction in ionic conductivity.

The values of ionic conductivities can be determined from *σ = d/AR*, in which *d* and *A* stand for the thickness and the area of the pellet, respectively, and *R* is the total resistance (*R*_2_), obtained above, and the results are given in Figure 2g. Among all the samples, the LAGP pellets sintered at 650 °C for 2 min exhibited the highest conductivity of 8.09 × 10^−5^ S cm^−1^ at RT. The plots of *log(σT)* vs. *1000/T* show a linear relation and fit well with the Arrhenius equation, *σ = A exp (E_a_/kT)*, in which *A*, *E_a_*, and *k* represents the pre-exponential factor, the activation energy for conduction, and the Boltzmann constant, respectively. As seen from the Arrhenius plots (Figure 2g), the slope of the 650 °C sample is the lowest. The activation energies (*E_a_*) are calculated from the rates of slopes, and the 650 °C sample’s *E_a_* is 0.293 eV. In Figure 2h, the XRD patterns of SPS pellets sintered at different temperatures are given. All of the LAGP pellets sintered at 600 °C to 700 °C maintained pure NASICON-type phase (JCPDS PDF No. 80-1922). When the temperature increased to 750 °C, the peak intensity increased at 26.4°, which corresponds to the (202) crystal plane of NASICON-type phase. This might be due to the reorientation of LAGP grains caused by the high pressure during the SPS process. For the whole heat treatment, the width of the diffraction peaks did not change, implying that the average crystallite size of LAGP remained unchanged.

LAGP SSEs sintered by SPS method could usually reach a very high density in a relatively low temperature (600–750 °C) and short time (2 min). The density of LAGP SSEs sintered at 600 °C was 3.219 g cm^−3^, as seen in Table 1. When the sintering temperature increased to 650 °C, the density increased up to 3.477 g cm^−3^. However, when the temperature further elevated to 700 °C and 750 °C, the density decreased to 2.495 and 2.430 g cm^−3^, respectively, most likely due to the formation of voids and microcracks in the pellets. The relatively density of LAGP SSEs was determined to be 90.4%, 97.6%, 70.1%, and 68.2%, for the LAGP pellets sintered at 600 °C, 650 °C, 700 °C, and 750 °C, respectively. Therefore, the SPS sintering temperature is a key parameter to determine the density and ionic conductivity of LAGP pellets.

**Figure 3:**
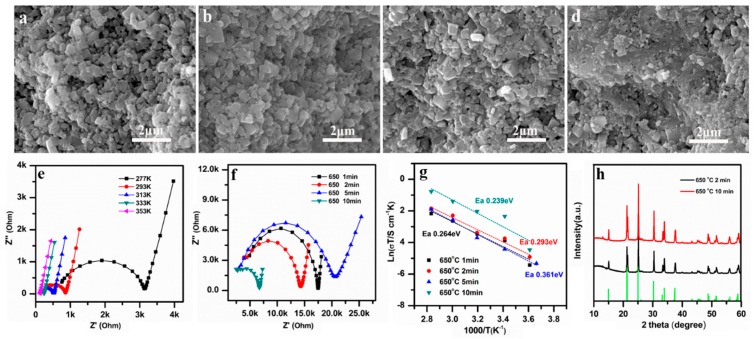
SEM images at cross sections of the LAGP pellets sintered at 650 °C for different sintering times: (**a**) 1 min, (**b**) 2 min, (**c**) 5 min, and (**d**) 10 min; (**e**) Nyquist plots of LAGP pellets (650 °C, 10 min) measured at different temperatures; (**f**) Nyquist plots, (**g**) Arrhenius plots, and (**h**) XRD patterns of the LAGP fabricated at 650 °C for 2 min and 10 min.

The SEM images of LAGP pellets subjected to 650 °C SPS sintering temperature for different times from 1 min, 2 min, 5 min, to 10 min are shown in Figure 3a–d, respectively. The microstructure of LAGP started to reorganize after 5 min of sintering, due to the melting that occurred at grain boundaries (Figure 3c). Most of the LAGP particles were fully joined after 10 min of sintering, and a highly densified structure was finally obtained (Figure 3d). SPS has a totally different densification mechanism compared with that of a conventional heating treatment. During the SPS process, Joule heating was introduced at the physical contact points of different particles, causing localized heating and possibly melting to facilitate the densification of the LAGP powders [41].

The EIS results for the samples obtained at 650 °C for 10 min are presented in Figure 3e, and the Nyquist plots of the samples sintered at 650 °C with different SPS times are shown in Figure 3f. It is obvious that the resistance of LAGP pellets decreased as the sintering time increased, as reflected by the reduction of the high-frequency semicircles in Figure 3f. When the sintering time reached 10 min, the LAGP pellets exhibited the highest ionic conductivity of 3.29 × 10^−4^ S cm^−1^ at RT. This result is consistent with the SEM microstructure observation (Figure 3d), which discloses the melting of grain boundaries. The high densification and well-jointed grain boundaries reduced the total resistance of the LAGP pellets. The *E_a_* calculation of the SPS samples processed at different times reveals that the 650 °C 10 min sample had a minimum *E_a_* of 0.239 eV, suggesting the lowest diffusion barrier for Li ions in SSEs. No impurity phases were detected in the XRD pattern for LAGP pellets sintered at 650 °C for 10 min.

**Figure 4:**
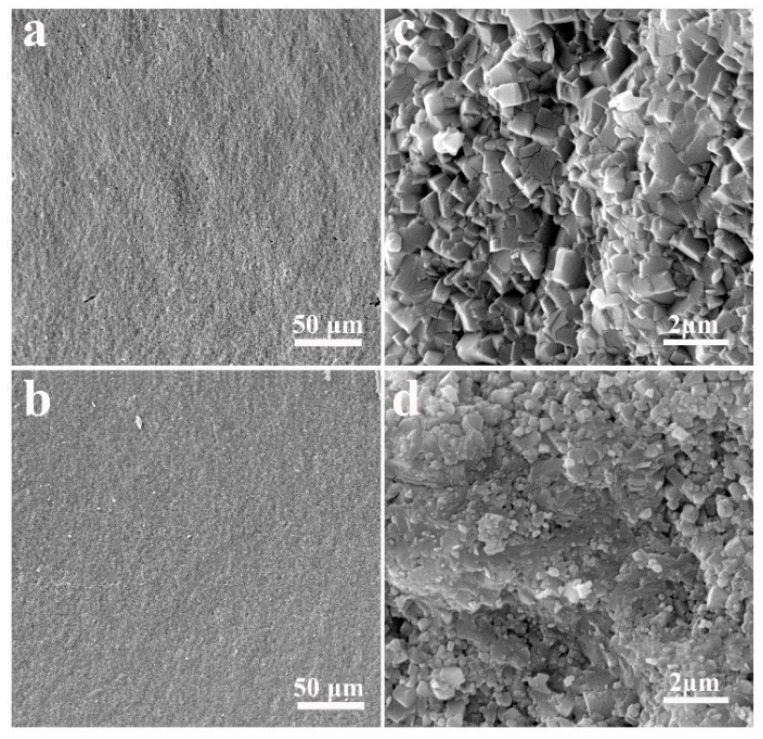
Cross-sectional SEM images of (**a**,**c**) conventionally sintered LAGP pellets and (**b**,**d**) SPS-sintered LAGP pellets.

In order to validate our sintering approach, it is necessary to investigate the impacts of the SPS compared with other conventional sintering methods. Therefore, LAGP pellets were also synthesized by a dry-pressing method, followed by heat treatment at 800 °C for 6 h, which was an optimal condition reported in a previous study [42]. Figure 4a,c show the SEM images of the samples obtained using a conventional sintering approach. It can be seen that the grain size of conventionally sintered LAGP pellet was in a range of 1.0–1.5 μm, which was much larger than that of SPS-sintered LAGP pellets (0.4–0.8 μm), as shown in Figure 4b,d.

Table 2 summarizes the correlations of preparation methods and conditions with the final density, relative density, ionic conductivity, and activation energy for LAGP SSEs. In conventional sintering approaches, high-temperature heat treatment is usually required to improve grain–grain interfaces and minimize grain-boundary resistance [31,42,43,44,45]. While low-temperature annealing leads to a low ionic conductivity, such as the glassy LAGP sample annealed at 500 °C, which is lower than its crystallization temperature [38]. This proved that the glassy phase of LAGP is not as good a Li-ion conductor as the crystallized one. However, two detrimental effects, i.e., the loss of lithium and formation of second phases, are usually concurrent for high-temperature heat treatment, and could cause the reduction of ionic conductivity in LAGP SSEs [31,42,43,44,45]. In our optimal SPS process, the sintering temperature was only 650 °C, which is much lower than other reported sintering methods, and yet the ionic conductivity remained high. At last but not least, LAGP pellets fabricated by SPS possess the highest relative density of 97.6% (Table 2).

## 4. Conclusions

In summary, highly densified LAGP pellets were fabricated by using SPS technique and exhibited high ionic conductivity at room temperature. The influence of SPS sintering temperatures (600–750 °C) and times (1–10 min) on the microstructure, density, and ionic conductivity of the LAGP pellets were studied in detail. It can be concluded that the optimal SPS condition for LAGP pellets was 650 °C and 10 min, and the synthesized LAGP pellets were dense with minimal visible porosity. The density of the LAGP pellets was as high as 3.477 g cm^−3^, a relative density of 97.6% and the ionic conductivity was 3.29 × 10^−4^ S cm^−1^, with a activation energy of 0.239 eV at RT. Sintering temperatures higher than 650 °C caused the formation of voids and microcracks in the LAGP pellets, negatively affecting the density and ionic conductivity. These results clearly demonstrate the potential of the SPS approach to synthesize solid electrolytes used in ASSLIBs, owing to the drastically reduced sintering temperature and time compared with that used in conventional sintering processes.

## Figures and Tables

**Table 1:** Comparison of the measured densities and the relative densities of LAGP sintered by SPS method at different temperatures.

SPS Sintering Temperature (°C)	600	650	700	650
Measured density (g cm^−3^)	3.219	3.477	2.495	2.430
Relative density (%) *	90.4	97.6	70.1	68.2

* Note: Relative density = measured density/theoretical density × 100. LAGP has a theoretical density of 3.5615 g cm^−3^.

**Table 2:** Summary of the density, relatively density, ionic conductivity, and activation energy for LAGP pellets fabricated using different methods.

SSEs	Preparation Method	Preparation Condition	Density (g cm^−3^)	Relative Density (%)	Ionic Conductivity (S cm^−1^)	Activation Energy (eV)	Ref.
Li_1.5_Al_0.5_Ge_1.5_(PO_4_)_3_	hot press + post-calcination	600 °C, 1 h, 200 MPa + 800 °C, 5h	3.33	93.5	1.64 × 10^−4^	0.299	[31]
Li_1.3_Al_0.3_Ge_1.7_(PO_4_)_3_	dry-pressing + post-calcination	5 min, 20 MPa + 750 °C, 5 h	3.18	89.3	3.4 × 10^−4^	0.330	[43]
Li_1.70_Al_0.61_Ge_1.35_ P_3.04_O_12.0_	cold press + sintering	800 °C, 6 h	3.02	84.8	2.3 × 10^−4^	0.320	[42]
Li_1.31_Al_0.42_Ge_1.52_P_3.09_O_12.1_	quenched glass piece + sintering	500 °C, 1 h	3.08	86.5	Too low to be measured	N/A	[42]
Li_1.5_Al_0.5_Ge_1.5_(PO_4_)_3_	melt-quench + post crystallization	800 °C, 8 h	N/A	N/A	5 × 10^−4^	0.280	[44]
Li_1.5_Al_0.5_Ge_1.5_(PO_4_)_3_	cold press + sintering	200 MPa + 850 °C, 5 h	N/A	N/A	2.99 × 10^−4^	N/A	[45]
Li_1.5_Al_0.5_Ge_1.5_(PO_4_)_3_	SPS	650 °C, 10 min, 100 MPa	3.477	97.6	3.29 × 10^−4^	0.239	This work

N/A: not available.

## Supplementary Materials

The following are available online at https://www.mdpi.com/2079-4991/9/8/1086/s1, Figure S1: Low-magnification SEM images of SPS pellets sintered for 2 min at different temperatures of (a) 650 °C, (b) 700 °C, and (c) 750 °C.
