# Supplementary material for: Spark Plasma Sintering of Lithium Aluminum Germanium Phosphate Solid Electrolyte and its Electrochemical Properties
Source: Nanomaterials (Basel). 2019 Jul 29;9(8):1086. doi: 10.3390/nano9081086 (PMC6722947; doi:10.3390/nano9081086)
Supplement: Supplementary file 1 [file nanomaterials-09-01086-s001.pdf]

nanomaterials

Supplementary Materials

Spark Plasma Sintering of Lithium Aluminum Germanium Phosphate Solid Electrolyte and its Electrochemical Properties

Hongzheng Zhu, Anil Prasad, Somi Doja, Lukas Bichler and Jian Liu *

School of Engineering, Faculty of Applied Science, The University of British Columbia, Kelowna, BC V1V 1V7 Canada

* Correspondence: Jian.liu@ubc.ca

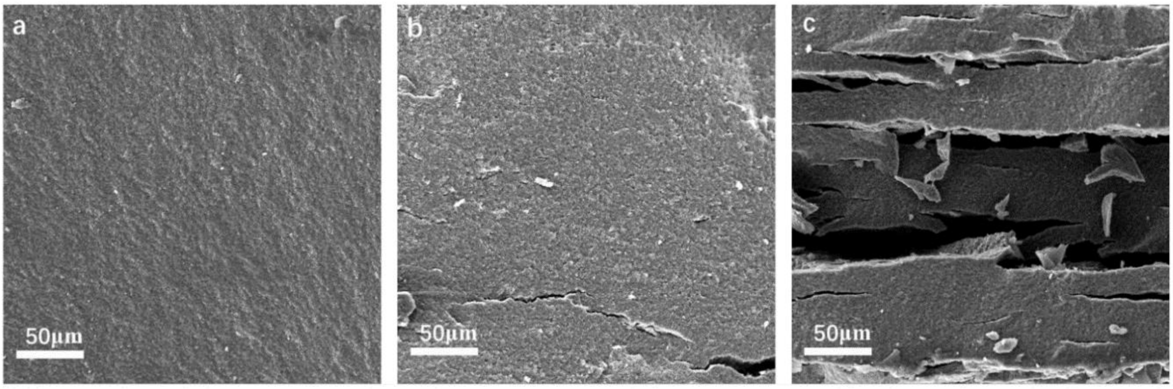

Figure S1. Low-magnification SEM images of SPS pellets sintered for 2 min at different temperatures of (**a**) 650 °C, (**b**) 700 °C, and (**c**) and 750 °C.
